# Combining machine learning and remote sensing-integrated crop modeling for rice and soybean crop simulation

**DOI:** 10.3389/fpls.2024.1320969

**Published:** 2024-02-12

**Authors:** Jonghan Ko, Taehwan Shin, Jiwoo Kang, Jaekyeong Baek, Wan-Gyu Sang

**Affiliations:** ^1^ Department of Applied Plant Science, Chonnam National University, Gwangju, Republic of Korea; ^2^ Crop Production and Physiology Division, National Institute of Crop Science, Wanju-gun, Jeollabuk-do, Republic of Korea

**Keywords:** crop, leaf area index, machine learning, modeling, remote sensing, rice, soybean, vegetation index

## Abstract

Machine learning (ML) techniques offer a promising avenue for improving the integration of remote sensing data into mathematical crop models, thereby enhancing crop growth prediction accuracy. A critical variable for this integration is the leaf area index (LAI), which can be accurately assessed using proximal or remote sensing data based on plant canopies. This study aimed to (1) develop a machine learning-based method for estimating the LAI in rice and soybean crops using proximal sensing data and (2) evaluate the performance of a Remote Sensing-Integrated Crop Model (RSCM) when integrated with the ML algorithms. To achieve these objectives, we analyzed rice and soybean datasets to identify the most effective ML algorithms for modeling the relationship between LAI and vegetation indices derived from canopy reflectance measurements. Our analyses employed a variety of ML regression models, including ridge, lasso, support vector machine, random forest, and extra trees. Among these, the extra trees regression model demonstrated the best performance, achieving test scores of 0.86 and 0.89 for rice and soybean crops, respectively. This model closely replicated observed LAI values under different nitrogen treatments, achieving Nash-Sutcliffe efficiencies of 0.93 for rice and 0.97 for soybean. Our findings show that incorporating ML techniques into RSCM effectively captures seasonal LAI variations across diverse field management practices, offering significant potential for improving crop growth and productivity monitoring.

## 1 Introduction

Crop models have traditionally been designed to simulate the impact of various environmental conditions on crop growth. These conventional models are invaluable for studying ideal growing conditions and guiding the best management practices (Lövenstein et al., 1992). However, they often rely on complex equations and parameters, which can result in discrepancies between the model’s predictions and actual field data (Maas, 1993; Ahuja et al., 2000). A well-calibrated model should accurately represent the growth and developmental stages of crops, provide precise yield predictions, and adapt its outputs based on relevant environmental variables (Ahuja et al., 2000).

Process-based crop models are particularly effective at simulating continuous crop development, growth, and yield using mathematical procedures and specific crop-related parameters. However, they struggle with complex spatial inputs and require extensive data on phenological and environmental variables throughout the growing season (Cao et al., 2021). These models frequently incorporate variables like the leaf area index (LAI) and various vegetation indices (VIs) derived from remote sensing (RS) data (Doraiswamy et al., 2005; Jeong et al., 2018; Nguyen et al., 2019; Shawon et al., 2020b). The use of the LAI and VIs helps minimize the effort and resources required to provide model inputs due to the benefits of RS that allows the observation of crop conditions. The benefits of this technique include real-time crop monitoring and the acquisition of various information depending on the radiometric sensors equipped with the instrument (Campbell and Wynne, 2011). RS techniques are helpful in scouting crop growth and its environments as they allow the observation of detailed information within a scene. RS methods can be applied to various aspects of monitoring and estimating crop conditions, including as an efficient estimation method of crop growth characteristics (Liu et al., 2022; Liu et al., 2023a). A weakness of RS is that it explains seasonal changes in crop conditions less than crop models. Integrating a crop model with RS information may enhance each other’s advantages and compensate for their weaknesses (Maas, 1993; Nguyen et al., 2019).

On the other hand, empirical regression methods offer a more simplified approach, relying on single or multiple regression techniques, but often fail to capture the complex, nonlinear relationships between environmental variables and crop performance (Nguyen et al., 2019; Sun et al., 2019).

A common challenge in crop models that integrate remote sensing data is the formulation of the LAI, which is often based on its linear relationship with VIs (Jin et al., 2018; Huang et al., 2019). These models face complications due to the dimensional differences between the 3-D LAI and 2-D VIs, variations across remote sensing platforms, and stage-specific differences in crop species (Huang et al., 2016; Nguyen et al., 2019). Recent advancements in machine learning (ML) techniques, such as the development of support vector machines (SVM), random forests (RF), one-dimensional convolutional neural networks (1D-CNN), and long-short-term memory (LSTM) networks, offer promising alternatives that may improve the accuracy of crop yield predictions (Cai et al., 2019; Van Klompenburg et al., 2020).

We believe that the integration of ML techniques can enhance the predictive accuracy of existing process-based crop models. Although initial efforts have incorporated crop model variables into ML frameworks, the comprehensive integration of ML algorithms into mathematical crop models has not been fully explored. Our study aims to fill this gap by introducing a novel methodology for LAI estimation using ML algorithms. In this study, we objectively compared the various ML (including deep neural network) regressors for simulating rice and soybean and then combined the selected one into the RSCM to evaluate the performance of the LAI simulation module. Specifically, we target rice (*Oryza sativa*) and soybean (*Glycine max*), for which accurate LAI estimation is critical yet challenging due to variable environmental and developmental factors.

## 2 Materials and methods

### 2.1 Field experiment data

Several datasets were used in this study to formulate ML and deep neural network (DNN) models and evaluate the selected ML scheme and the ML-combined remote sensing-integrated crop model (RSCM) performance. To develop an ML or a DNN scheme for the relationships between the LAI and VIs of rice and soybean (**Supplementary Figures 1** and **2**), we used rice data (n = 552) obtained with proximal and remote sensing methods from 2011 to 2014 (Yeom et al., 2021) and soybean data (n = 556) obtained with proximal sensing methods from 2017 to 2018 (Shawon et al., 2020a).

The model evaluation datasets were obtained from the Chonnam National University (CNU) experimental field (35°10’ N, 126°53’ E), Gwangju, and the National Institute of Crop Science (NICS) experimental field (35°50’ N, 127°02’ E), Wanju, Jeonbuk province, from 2021 to 2022. The rice cultivar ‘Shindongjin’ was cultivated at the CNU field (~1,400 m^2^), which was divided into three different nitrogen (N) treatments (no N, heavy N, and full N), and at the NICS field (~1,200 m^2^), divided into two N treatments (no N and full N). The soybean cultivar ‘Daepung’ was grown at the NICS field (~2,000 m^2^) with three N treatments (0 kg ha^−1^, 24 kg ha^−1^, and 48 kg ha^−1^). Crop management practices during the seasons followed the standard NICS cultivation procedures for each crop. Weather conditions at the NICS study site were automatically recorded using a mechanical MetPRO (Campbell, Logan, UT, USA) weather station. Weather data for the CNU study site were obtained from the Open MET Data Portal (https://data.kma.go.kr, accessed on September 14, 2023) of the Korea Meteorological Administration (KMA). The KMA weather station is adjacent (within ~1 km) to the experimental field. From 20 May to 20 October, the daily average mean temperature, solar radiation, and precipitation at CNU were 24.21°C, 17.04 MJ m^−2^ d^−1^, and 5.67 mm d^−1^, respectively, during the 2021 season and 24.39°C, 17.28 MJ m^−2^ d^−1^, and 3.47 mm d^−1^, respectively, during the 2022 season. During the same period at NICS, the daily average mean temperature, solar radiation, and precipitation were 23.99°C, 16.04 MJ m^−2^ d^−1^, and 7.22 mm d^−1^, respectively, in 2021 and 23.92°C, 16.31 MJ m^−2^ d^−1^, and 5.08 mm d^−1^, respectively, in 2022.

The LAI and canopy reflectance data for rice and soybean were measured using an LI-2200C (LiCor, Inc., Lincoln, NE, USA) and a hand-held multispectral radiometer, MSR16R (CropScan, Inc., Rochester, MN, USA). An LAI-2200C can accurately measure canopy LAI in diffuse sunlight using light-scattering correction. The MSR16R had 16 waveband filters in the 450−1,750 nm region, equipped with upward and downward sensors (http://www.cropscan.com/, accessed on January 21, 2024). This design allows for simultaneously measuring both incoming and reflected radiation, providing valid reflectance readings in lightly cloudy conditions with incident irradiance down to approximately 300 W m^−2^. The canopy reflectance data were obtained during the crop growing seasons at the study sites, six times in 2021 on day of year (DOY) 194, 210, 224, 238, 259, and 273 and five times in 2022 on DOY 203, 230, 244, 263, and 280. All field measurement operations to determine crop canopy reflectance were conducted in the clear sky within an hour of the local solar noon (12:40 pm KST) to minimize potential influences of perspective on the remote imaging of plants.

The canopy reflectance data were arithmetically transformed to get the VIs of interest for simulating LAI. These VIs included the modified triangle vegetation index 1 (MTVI1; Equation 1) (Haboudane et al., 2004), normalized vegetation index (NDVI; Equation 2) (Rouse et al., 1974), optimized soil adjusted vegetation index (OSAVI; Equation 3) (Rondeaux et al., 1996), and renormalized difference vegetation index (RDVI; Equation 4) (Roujean and Breon, 1995). The VI equations were determined using reflectance values at 560 nm (*R*_560_), 660 nm (*R*_660_), and 800 nm (*R*_800_):


(1) $$\text{MTVI}1=1.2({\text{R}}_{800}-{\text{R}}_{660})-2.5({\text{R}}_{660}+{\text{R}}_{560})$$

(2) $$NDVI=\left({\text{R}}_{800}-{\text{R}}_{660}\right)/\left({\text{R}}_{800}+{\text{R}}_{660}\right)$$

(3) $$OSAVI=\left({\text{R}}_{800}-{\text{R}}_{660}\right)/\left({\text{R}}_{800}+{\text{R}}_{660}+0.16\right)$$

(4) $$RDVI=\left({\text{R}}_{800}-{\text{R}}_{660}\right)/\sqrt{\left({\text{R}}_{800}+{\text{R}}_{660}\right)}$$

The relationships between the LAI and the VIs of rice (**Supplementary Figure 1**) and soybean (**Supplementary Figure 2**) were investigated to determine the optimal LAI estimation algorithms out of the various ML regression models described in the following subsection.

### 2.2 ML and DNN models

In this study, we explored various ML algorithms, including polynomial regression, ridge regression, least absolute shrinkage and selection operator (LASSO) regression, support vector regression (SVR), RF, extra trees (ET), and gradient boosting (GB) and its variants, histogram-based gradient boosting (HGB), extreme gradient boosting (XGB), and light gradient boosting machine (LightGBM). These algorithms are available through the Python-based *scikit-learn* library. In addition, we utilized the feed-forward DNN, implemented using the *Keras* framework (https://keras.io, accessed on September 14, 2023) in Python (https://www.python.org, accessed on September 14, 2023).

Polynomial Regression extends the capabilities of least-squares linear regression by applying an n^th^-degree polynomial, improving performance over standard linear regression. Ridge and LASSO regression methods further optimize performance by incorporating l2 and l1 norms to reduce overfitting (Diebold and Shin, 2019; Emami Javanmard et al., 2021).

The SVR method defines a specific error tolerance and identifies an optimal hyperplane in a higher-dimensional space, providing advantages in classification and prediction tasks. However, it is computationally intensive, and the outcomes are less easily interpretable (Khosla et al., 2020).

The RF algorithm employs an ensemble of decision trees for better generalization and is relatively robust against overfitting, and ET adds an element of randomness to each decision tree split, thereby reducing bias and variance (Wang et al., 2019). Unlike RF, ET does not utilize bootstrap sampling. The GB algorithm and its advanced forms (i.e., HGB, XGB, and LightGBM) augment performance by focusing on training speed and reducing overfitting (Ustuner and Balik Sanli, 2019).

For the DNN model, we increased predictive accuracy by adding multiple hidden layers between the input and output (**Supplementary Figure 3**). Despite its high performance, the DNN model must be revised for interpretation. It should be noted that traditional ML models may outperform DNNs when the dataset is small (Jeong et al., 2022b).

The dataset was split into training and testing subsets using an 80:20 ratio through the *scikit-learn* package. All ML and DNN models were fine-tuned to identify optimal hyperparameters. For ridge and LASSO regressions, alpha values of 0.1 and 0.01 were chosen based on a grid search. The DNN model employed a rectified linear unit (ReLU) activation function consisting of six fully connected layers ranging from 100 to 1,000 units (**Supplementary Figure 3**). A dropout rate of 0.17 and the “Adam” optimizer with a learning rate of 0.001 were applied over 1,000 epochs, with a batch size of 100.

### 2.3 Process-based crop model

**Figure 1:**
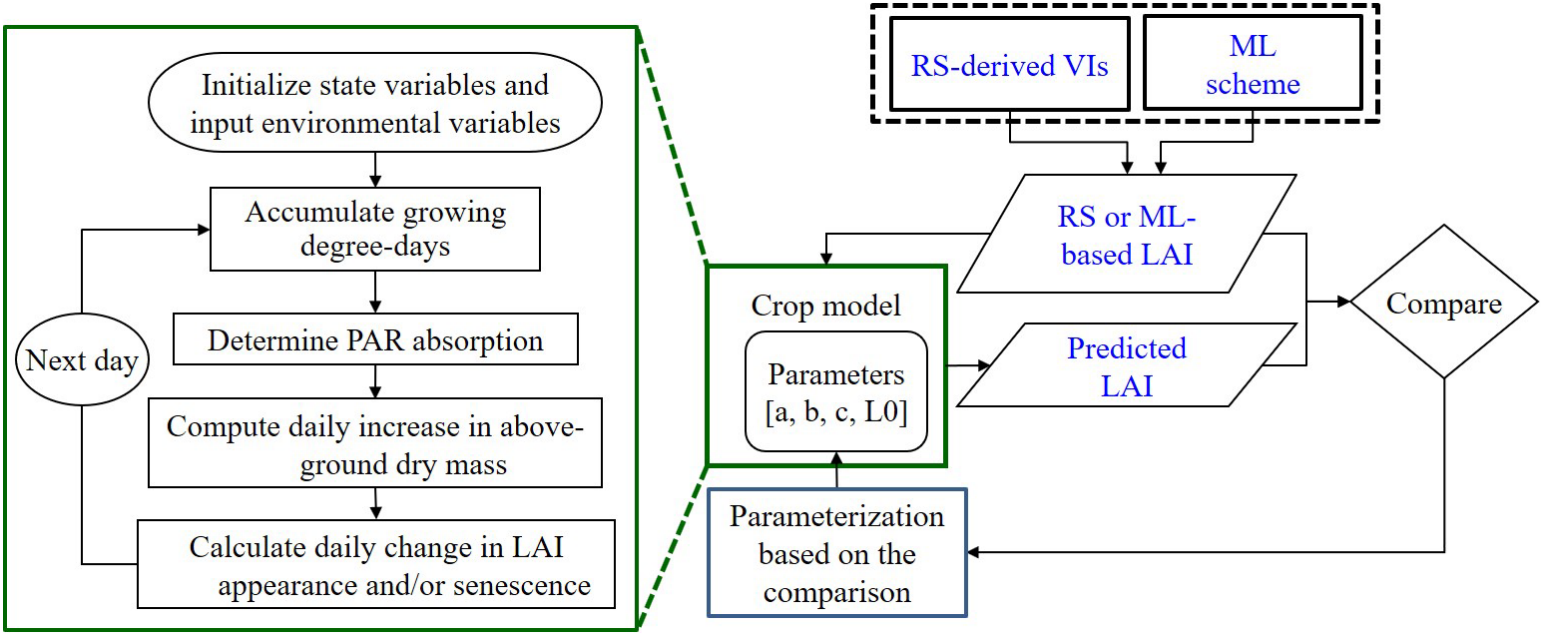
Diagrammatic representation of the remote sensing (RS)-integrated crop model combined with a machine learning (ML) method for predicting the leaf area index (LAI) based on vegetative indices (VIs). Adapted from Nguyen et al., 2019. PAR stands for photosynthetically active radiation.

This study employed an RSCM augmented with ML to simulate crop growth (specifically LAI), as depicted in **Figure 1**. Following an evaluation of various ML and DNN regressors, detailed in the subsequent subsection, we integrated a selected ML algorithm into the RSCM framework. This ML integration was designed to enhance the regression methods for assessing the relationship between remotely sensed VIs and LAI.

The RSCM is a process-oriented model (**Table 1**) crafted to assimilate data collected through remote sensing, enabling researchers to simulate and scrutinize potential crop development (Nguyen et al., 2019). Four mathematical procedures were employed in the crop modeling: (1) daily change in growing degree days (GDD), (2) absorption of incident solar radiation, (3) daily increase in above-ground dry mass, and (4) daily LAI increase. The RSCM uses daily maximum and minimum temperatures and solar radiation as input variables to determine GDD and solar radiation absorption by the crop canopy. Crop-specific coefficients were adopted from those obtained earlier by Nguyen et al. (2019) for rice and Shawon et al. (2020a) for soybean (**Table 2**).

**Table 1:** Equations applied in the remote sensing-integrated crop model.

Equations	Variable descriptions
ΔD = MAX [T − T_b_, 0]	ΔD, daily change in growing degree days (GDD); T, daily mean temperature; T_b_, crop-specific base temperature
Q = β ∙ R ∙ (1 − e^−k∙LAI^)	Q, absorption of incident solar radiation (R); β, fraction of R (i.e., 0.45); k, crop-specific light extinction coefficient; LAI, leaf area index
ΔM = ε ∙ Q	ΔM, daily increase in above-ground dry mass; ε, radiation use efficiency
ΔL = ΔM ∙ P_1_ ∙ S	ΔL, daily LAI increase; P1, fraction of ΔM allocated to new leaves; S, specific leaf area
P_1_ = Max [1 − a ∙ e^b∙D^,0]	P_1_, dimensionless leaf allocation function; a and b, parameters controlling magnitude and shape of the process; D, cumulative GDD

**Table 2:** Parameter values used for the remote sensing-integrated crop model.

Symbol	Description	Unit	Value	
			Rice	Soybean
ε	Radiation use efficiency	g MJ^−1^	3.49	1.65
*k*	Light extinction coefficient	na	0.60	0.71
*S*	Specific leaf area	m^2^ g^−1^	0.016	0.017
*T*_b_	Base temperature	°C	12.0	10.0

The RSCM can incorporate remote sensing information for its in-season calibration process (Maas, 1993). In this process, predicted LAI metrics are juxtaposed with their observed counterparts. The calibration uses four specific parameters—*L_0_, a, b*, and *c*—to model crop growth dynamics based on optimizing the LAI through the Powell procedure (Press et al., 1992). Moreover, Bayesian methods can be applied to these parameters for calibration, leveraging prior distributions inferred from previous research to yield acceptable parameter values (Ko et al., 2015; Nguyen et al., 2019). In this study, we employed exponential regressions to determine the LAI and VI relationships of rice and soybean (**Supplementary Table 1**).

All the parameters were objectively reparametrized to match the predicted LAI with the RS- or ML-based LAI. The converged parameter values after the in-season calibration are shown for rice in **Supplementary Table 2** and for soybean in **Supplementary Table 3**. For this study, we used consistent initial settings and parameters to fine-tune the RSCM specifically for rice and soybean crop modeling (i.e., *L_0 =_* 0.2, *a =* 3.25 × 10^−1^, *b* = 1.25 × 10^−3^, and *c* = 1.25 × 10^−3^).

### 2.4 Statistical evaluation of simulation performances

Model assessments were achieved by comparing the simulated or predicted values to the observed values in the testing subset. For the statistical evaluation, we employed the root mean square error (RMSE; Equation 5), the mean absolute error (MAE; Equation 6), and the Nash–Sutcliffe efficiency (NSE; Equation 7) (Nash and Sutcliffe, 1970):


(5) $$RMSE={\left[\frac{1}{\text{n}}\sum_{\text{i}=1}^{\text{n}}{\left({\text{S}}_{\text{i}}-{\text{O}}_{\text{i}}\right)}^{2}\right]}^{0.5}$$

(6) $$MAE=\frac{\sum_{\text{i}=1}^{\text{n}}S_{i}-O_{i}}{\text{n}}$$

(7) $$NSE=1-\frac{\sum_{\text{i}=1}^{\text{n}}{\left(S_{i}-O_{i}\right)}^{2}}{\sum_{\text{i}=1}^{\text{n}}{\left(O_{i}-\bar{\text{O}}\right)}^{2}}$$

where *S_i_* represents the simulated value at measurement point *i* and *n*, *O_i_*, and $\bar{O}$ represent the total number of data points, the observed value at measurement point *i*, and the mean of the observed values, respectively. The RMSE and the MAE quantify the average variance between the simulated and the observed values on the metric scale of the respective model, and the NSE evaluates model performance efficiency with an index ranging from −∞ to one (unitless). A suitable fit between the simulated and the observed data is indicated by RMSE and MAE values close to 0 and NSE values close to 1.0.

## 3 Results

In this study, we successfully developed ML models to estimate the LAI for two significant staple crops: rice and soybean. We tested these models across two different study sites with varying N treatments by integrating them into the RSCM scheme.

### 3.1 LAI estimation using ML and DNN models

The test scores for the ten selected ML regression models ranged from 0.783 to 0.859 for rice and from 0.770 to 0.889 for soybean (**Table 3**). The ET regressor outperformed other algorithms, achieving test scores of 0.859 and 0.889 for rice and soybean, respectively. We also found that most other ML algorithms performed comparably to the ET regressor.

**Table 3:** Training and test scores for the regression analyses between leaf area index and vegetation indices for rice and soybean using 10 machine learning models.

Regressor*	Rice		Soybean	
	Training score	Test score	Training score	Test score
Polynomial Linear	0.776	0.783	0.852	0.770
Ridge	0.823	0.834	0.852	0.773
Lasso	0.781	0.791	0.776	0.741
Support Vector	0.823	0.837	0.853	0.791
Random Forest	0.978	0.844	0.982	0.882
Extra Trees*	**0.999**	**0.859**	**0.999**	**0.889**
Gradient Boosting	0.959	0.831	0.966	0.872
Histogram-based Gradient Boosting	0.931	0.851	0.938	0.839
XGBoost	0.999	0.842	0.999	0.878
LightGBM	0.925	0.848	0.929	0.836

* The bold characters represent the select regressor and scores.

**Figure 2:**
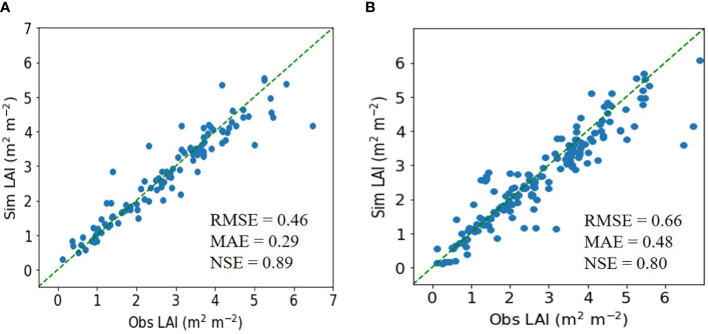
Simulated (Sim) versus observed (Obs) leaf area index (LAI) values for rice in the tests of the **(A)** extra trees and **(B)** deep neural network regressors. The diagonal dashed reference lines represent the 1:1 relationship. RMSE, MAE, and NSE stand for root mean square error, mean absolute error, and Nash–Sutcliffe efficiency.

In testing the ET regressor, the RMSE was 0.46 m^2^ m^−2^, the MAE was 0.29 m^2^ m^−2^, and the NSE was 0.89 for rice (**Figure 2**). These metrics were superior to those from the DNN model.

**Figure 3:**
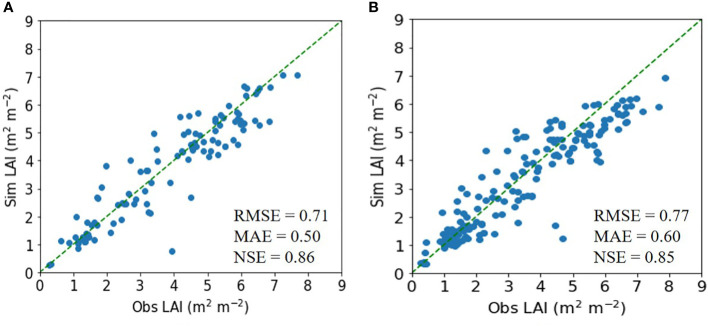
Simulated (Sim) versus observed (Obs) leaf area index (LAI) values for soybean in the tests of the **(A)** extra trees and **(B)** deep neural network regressors. The diagonal dashed reference lines represent the 1:1 relationship. RMSE, MAE, and NSE stand for root mean square error, mean absolute error, and Nash–Sutcliffe efficiency.

Similarly, for soybean, the ET model achieved an RMSE of 0.71 m^2^ m^−2^, an MAE of 0.50 m^2^ m^−2^, and an NSE of 0.86, outperforming the DNN model (**Figure 3**).

### 3.2 Evaluation and application of the ML model

**Figure 4:**
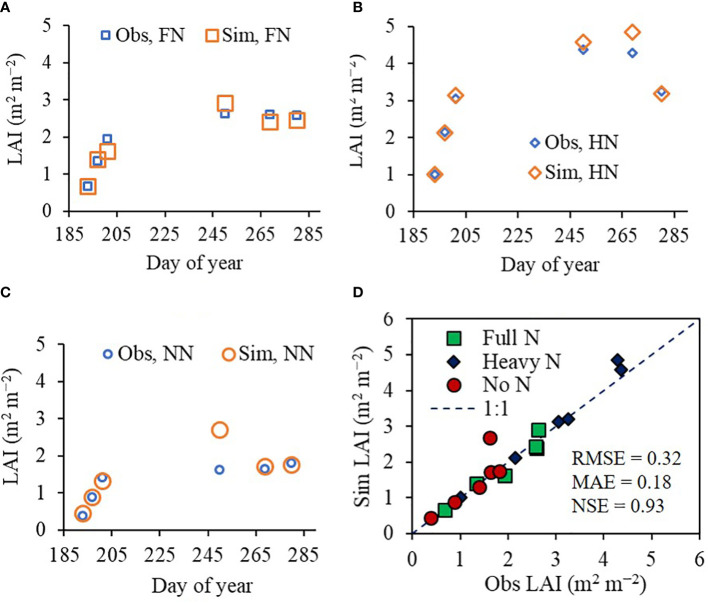
Simulated (Sim) versus observed (Obs) leaf area index (LAI) values of rice grown with different nitrogen (N) treatments at the Chonnam National University’s experimental field in 2022. Seasonal variations in the Sim and Obs LAI values with **(A)** full nitrogen (FN), **(B)** heavy nitrogen (HN), and **(C)** no nitrogen (NN) treatments are shown along with **(D)** a comparison between the Sim and Obs LAI values including all three N treatments. The diagonal dashed reference line in **(D)** represents the 1:1 relationship, and the root mean square error (RMSE), mean absolute error (MAE), and Nash–Sutcliffe efficiency (NSE) values for the predictions are displayed.

We demonstrated that the ET model could accurately simulate seasonal LAI variation for rice under different N treatments. The model was tested in two different fields during 2022: the CNU experimental field and the NICS experimental field. The LAI values simulated using the CNU field conditions agreed with the corresponding observed LAI values in the field, achieving an RMSE of 0.32 m^2^ m^−2^, an MAE of 0.18 m^2^ m^−2^, and an NSE of 0.93 (**Figure 4**). In the equivalent model evaluation using the NICS field dataset (**Supplementary Figure 4**), the simulated LAI values again matched with the observed values, with an RMSE of 0.20 m^2^ m^−2^, MAE of 0.14 m^2^ m^−2^, and NSE of 0.85.

**Figure 5:**
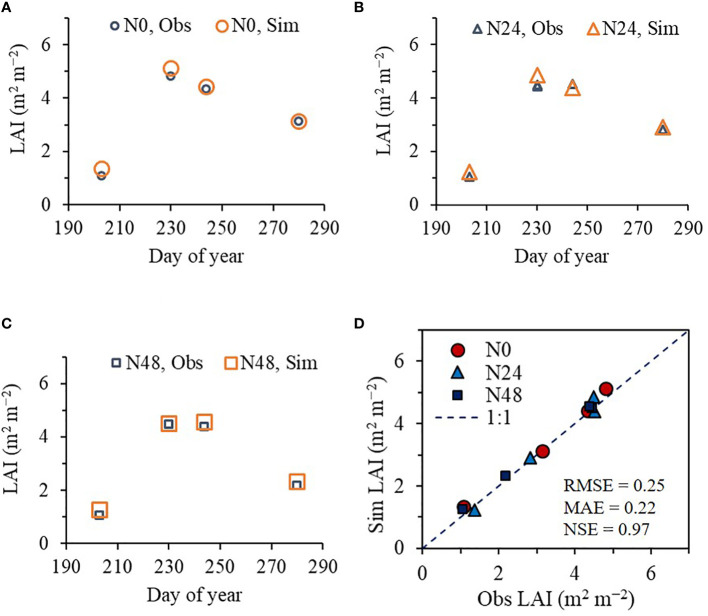
Simulated (Sim) versus observed (Obs) leaf area index (LAI) values of soybean grown with different nitrogen (N) treatments at the National Institute of Crop Science’s experimental fields in 2022. Seasonal variations in the Sim and Obs LAI values with the nitrogen treatments at **(A)** 0 kg ha^−1^ (N0), **(B)** 24 kg ha^−1^ (N24), and **(C)** 48 kg ha^−1^ (N48) are shown along with **(D)** a comparison between the Sim and Obs LAI values including all three N treatments. The diagonal dashed reference line in **(D)** represents the 1:1 relationship, and the root mean square error (RMSE), mean absolute error (MAE), and Nash–Sutcliffe efficiency (NSE) values for the predictions are displayed.

As with rice, the ET model effectively predicted seasonal variation in soybean LAI at the NICS experimental field in 2022. The predicted LAI values agreed with the corresponding observed LAI values, with an RMSE of 0.25 m^2^ m^−2^, MAE of 0.22 m^2^ m^−2^, and NSE of 0.97 (**Figure 5**).

**Figure 6:**
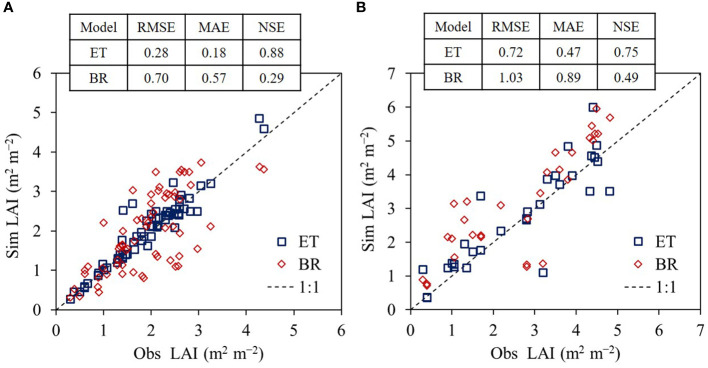
Comparison of extra trees (ET) and Bayesian-based regression (BR) models in leaf area index (LAI) simulation performances for rice **(A)** and soybean **(B)**. The modeling capabilities were investigated with root mean square error (RMSE), mean absolute error (MAE), and Nash–Sutcliffe efficiency (NSE) between simulated (Sim) and observed (Obs) LAI values using the evaluation data applied in this study.

We found the ET regressor outperformed the Bayesian-based regression (BR) model in the both crops (**Figure 6**). Simulated rice LAI values agreed with the observed rice LAI values with an RMSE of 0.28, MAE of 0.18, and NSE of 0.88 for ET compared with an RMSE of 0.70, MAE of 0.57, and NSE of 0.29 for the BR model. In soybean, simulated LAI values matched the observed LAI values with an RMSE of 0.72, MAE of 0.47, and NSE of 0.75 for ET compared with an RMSE of 1.03, MAE of 0.89, and NSE of 0.49 for the BR model.

**Figure 7:**
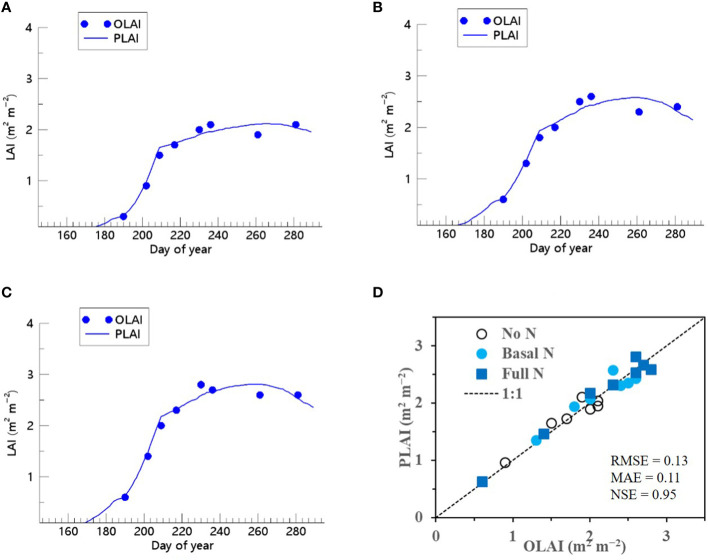
Predicted (PLAI) versus observed (OLAI) leaf area index (LAI) valuesof rice grown with different nitrogen (N) treatments at the Chonnam National University’s experimental fields in 2021. Seasonal variations in LAI values with **(A)** no N, **(B)** basal N, and **(C)** full N treatments are shown along with **(D)** a comparison between PLAI and OLAI including all three N treatments. The diagonal dashed reference line in **(D)** represents the 1:1 relationship, and the root mean square error (RMSE), mean absolute error (MAE), and Nash–Sutcliffe efficiency (NSE) values for the predictions are displayed.

**Figure 8:**
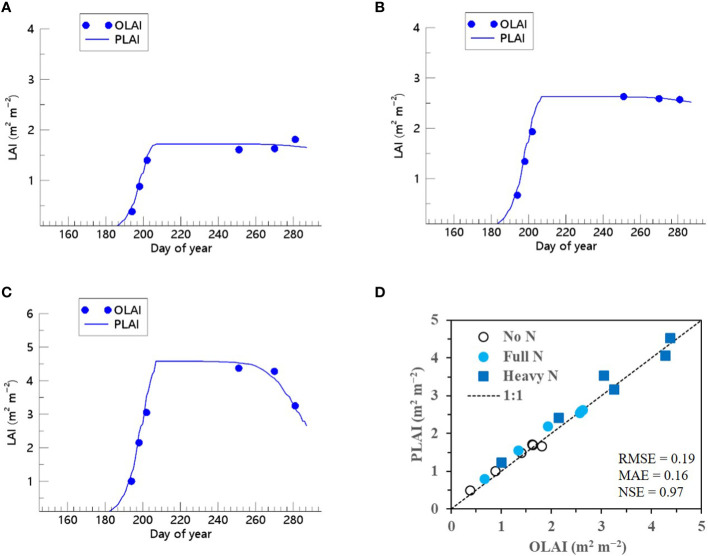
Predicted (PLAI) versus observed (OLAI) leaf area index (LAI) valuesof rice grown with different nitrogen (N) treatments at the Chonnam National University’s experimental fields in 2022. Seasonal variations in LAI values with **(A)** no N, **(B)** full N, and **(C)** heavy N treatments are shown along with **(D)** a comparison between PLAI and OLAI including all three N treatments. The diagonal dashed reference line in **(D)** represents the 1:1 relationship, and the root mean square error (RMSE), mean absolute error (MAE), and Nash–Sutcliffe efficiency (NSE) values for the predictions are displayed.

We showed that the RSCM assimilated with the ET regressor could closely predict seasonal variations in rice LAI under different N treatments at both the CNU (**Figures 7** and **8**) and NICS (**Supplementary Figures 5** and **6**) fields during 2021 and 2022. The RSCM model attained an RMSE of 0.13, MAE of 0.11, and NSE of 0.95 in 2021 and an RMSE of 0.19, MAE of 0.16, and NSE of 0.97 in 2022 at the CNU field (**Figures 7** and **8**). At the NICS fields, the RSCM model attained an RMSE of 0.05, MAE of 0.04, and NSE of 0.99 in 2021 and an RMSE of 0.09, MAE of 0.07, and NSE of 0.98 in 2022 (**Supplementary Figures 5** and **6**).

**Figure 9:**
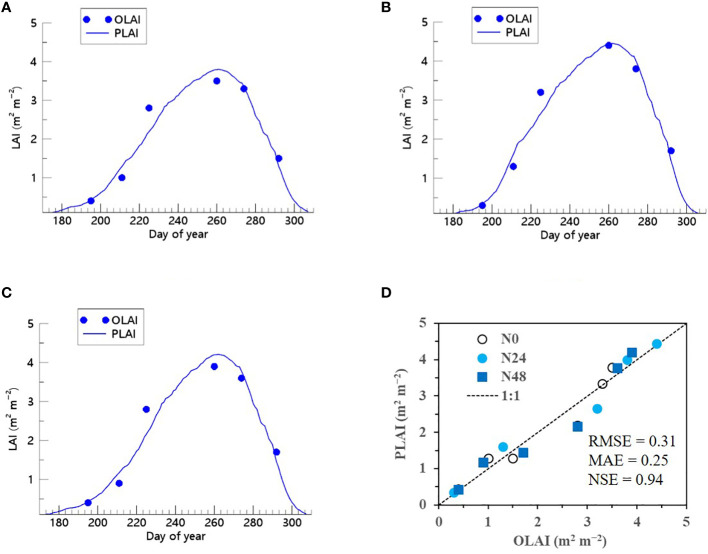
Predicted (PLAI) versus observed (OLAI) leaf area index (LAI) valuesof soybean grown with different nitrogen (N) treatments at the National Institute of Crop Science’s experimental field in 2021. Seasonal variations in LAI values with **(A)** no N, **(B)** 24 kg N ha^−1^, and **(C)** 48 kg N ha^−1^ treatments are shown along with **(D)** a comparison between PLAI and OLAI including all three N treatments. The diagonal dashed reference line in **(D)** represents the 1:1 relationship, and the root mean square error (RMSE), mean absolute error (MAE), and Nash–Sutcliffe efficiency (NSE) values for the predictions are displayed.

**Figure 10:**
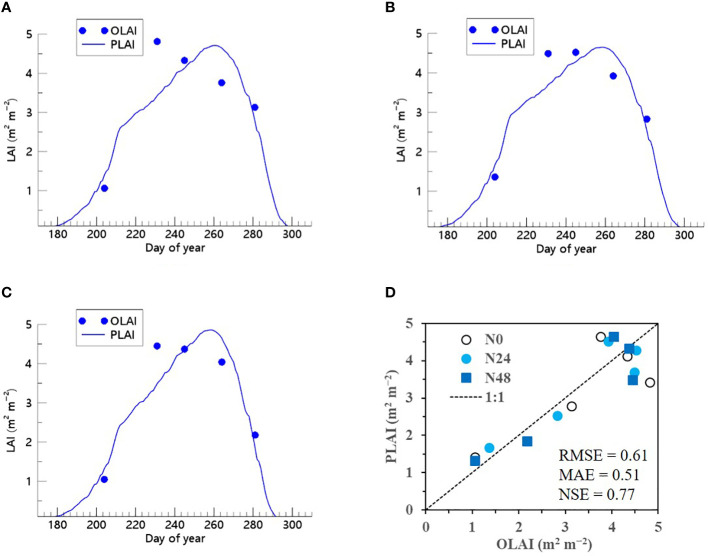
Predicted (PLAI) versus observed (OLAI) leaf area index (LAI) valuesof soybean with different nitrogen (N) treatments at the National Institute of Crop Science’s experimental field in 2022. Seasonal variations in LAI values with **(A)** no N, **(B)** 24 kg N ha^−1^, and **(C)** 48 kg N ha^−1^ treatments are shown along with **(D)** a comparison between PLAI and OLAI including all three N treatments. The diagonal dashed reference line in **(D)** represents the 1:1 relationship, and the root mean square error (RMSE), mean absolute error (MAE), and Nash–Sutcliffe efficiency (NSE) values for the predictions are displayed.

We also demonstrated that when the ET algorithm was incorporated into the RSCM, it could closely replicate seasonal variations in soybean LAI across multiple years and N treatment conditions (**Figures 9** and **10**). The RSCM model produced an RMSE of 0.31, MAE of 0.25, and SE of 0.94 in 2021 and an RMSE of 0.61, MAE of 0.51, and NSE of 0.77 in 2022.

## 4 Discussion

Our research explored the advantages of integrating ML and DNN techniques into existing process-based crop models. This integration aims to address the complex equations and parameters that often result in discrepancies between simulated and actual field data. By combining traditional crop modeling with advanced ML and DNN methods, we achieved a higher level of predictive accuracy and reliability for simulating the LAI of rice and soybean crops.

Our study found that the ET regressor was the most effective ML model for simulating LAI values with the 0.89 NSE test score for rice and the 0.86 NSE test score for soybean, surpassing the DNN-based model and the Bayesian-based regression method (see **Figure 6**). We hypothesize that the improved accuracy of the ET regressor may be due to a nonlinear relationship between VIs and LAI. This is similar to a recent report on the relationship between VI and aboveground biomass by Liu et al. (2023b). These findings corroborate recent studies (Jeong et al., 2022a; Shin et al., 2022) but contradict earlier research suggesting the superiority of DNN techniques (Bui et al., 2020; Sahoo et al., 2020). This discrepancy may highlight the limitations of our dataset’s scope and specific characteristics in determining simulation effectiveness. It is conceivable that applying a more diverse dataset in future research could potentially yield results affirming the efficacy of DNN-based regressors.

We also evaluated the revised RSCM, which integrates both proximal and RS data. This innovative framework successfully predicts spatiotemporal variations in rice and soybean growth at the field scale. Incorporating RS data streamlines data collection and enhances the model’s simulation performance, making it applicable across different geographic regions. However, limitations, such as the partial capture of RS data, still exist and may lead to forecasting inaccuracies.

Incorporating RS data into process-based crop models, specifically within the framework of the RSCM, confers several notable benefits. Firstly, this approach significantly streamlines the range of input parameters and variables required. Rather than relying on a cumbersome array of factors, the model accepts existing remotely sensed and proximal data as pivotal elements for depicting the environmental context accurately. This has the effect of simplifying the data acquisition process, making it more manageable and less resource-intensive since the current methodology can be directly applied to those using the other RS platforms. These include operational optical satellite sensors, e.g., Jeong et al. (2022a) and remote-controlled aerial systems, e.g., Shin et al. (2022). Secondly, integrating RS data directly translates to enhanced simulation performance in the RSCM system. Including this data enables the model to generate more accurate, reliable, and nuanced forecasts of crop growth patterns and yields, thereby improving its utility and predictive capabilities. Thirdly, this methodology allows for the assimilation of RS information sourced from a diverse array of operational optical sensors with differing spatial resolutions. These sensors could be from a variety of platforms, including those on satellites (Yeom et al., 2018; Nguyen et al., 2019; Yeom et al., 2021) as well as those mounted on remotely piloted aerial systems (Jeong et al., 2018). This flexibility dramatically enriches the dataset that the RSCM can draw from, leading to more comprehensive and holistic analyses. Lastly, the adaptability of the RSCM framework makes it universally applicable across different geographical locales, even in regions where data might be sparse or in physically inaccessible areas (Yeom et al., 2018; Jeong et al., 2020). The only requisite is the availability of satellite imagery, which is generally accessible globally.

Despite these advantages, it is worth noting that the RSCM optimization technique has limitations. Among these are the incomplete or partial capture of RS data and the potential for restricted proximal data during the crop’s growing cycle. These constraints may result in discrepancies between predicted outcomes and actual observations and, thus, inaccuracies in crop growth and productivity forecasting.

## 5 Conclusion

This study evaluated the ability of multiple ML models to simulate LAIs using VIs from proximal data sources and found the ET model to be the most effective for both rice and soybean crops. Our findings demonstrate the viability of integrating ML and DNN methodologies into a process-based crop model that uses RS data. These integrated models can improve crop growth and productivity monitoring. Although this research lays a foundation for integrating ML into the RSCM framework, further work is needed to extend these methodologies, particularly in simulating other variables like carbon and water fluxes.

## Data availability statement

The original contributions presented in the study are included in the article/**Supplementary Material**. Further inquiries can be directed to the corresponding author.

## Author contributions

JKo: Conceptualization, Funding acquisition, Project administration, Software, Supervision, Writing – original draft, Writing – review & editing. TS: Data curation, Investigation, Methodology, Resources, Writing – review & editing. JKa: Data curation, Investigation, Methodology, Resources, Writing – review & editing. J-KB: Data curation, Investigation, Methodology, Resources, Writing – review & editing. W-KS: Data curation, Funding acquisition, Investigation, Methodology, Project administration, Resources, Writing – review & editing.
