# Supplementary material for: Combining machine learning and remote sensing-integrated crop modeling for rice and soybean crop simulation
Source: Front Plant Sci. 2024 Feb 12;15:1320969. doi: 10.3389/fpls.2024.1320969 (PMC10894942; doi:10.3389/fpls.2024.1320969)
Supplement: Supplementary file 1 [file DataSheet_1.docx]

Supplementary material

# **Supplementary Figures**


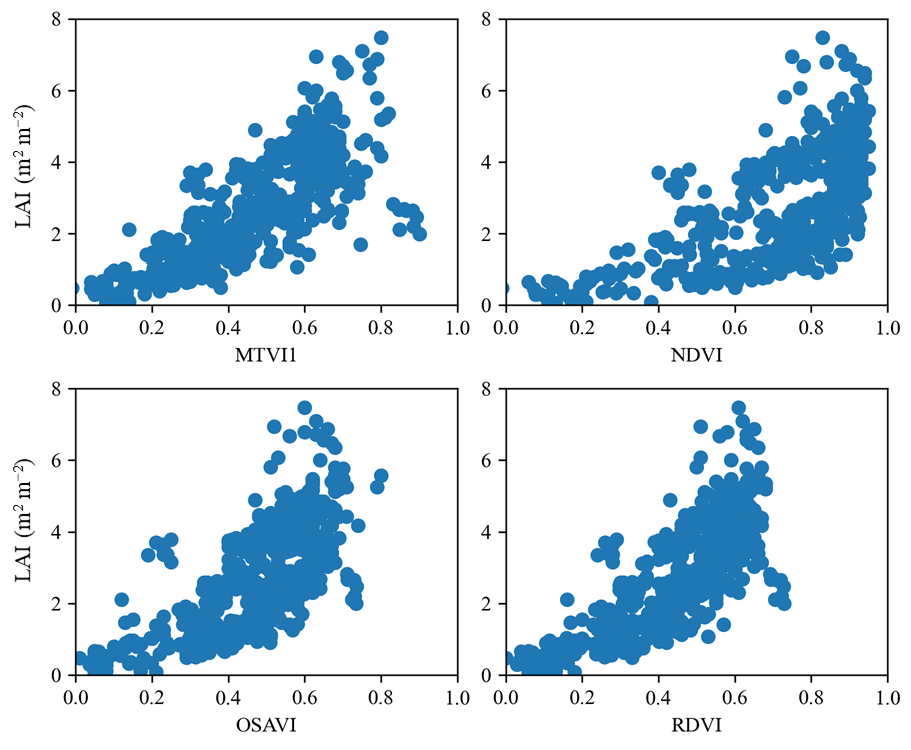


**Supplementary Figure 1.** Leaf area index (LAI) versus the vegetation indices (VIs) selected for rice (n = 552) for the regression analyses in Table 1. The best regressor was Extra Trees, with a test score of 0.859. The VIs include the modified triangle vegetation index 1 (MTVI1), normalized vegetation index (NDVI), optimized soil adjusted vegetation index (OSAVI), and renormalized difference vegetation index (RDVI).


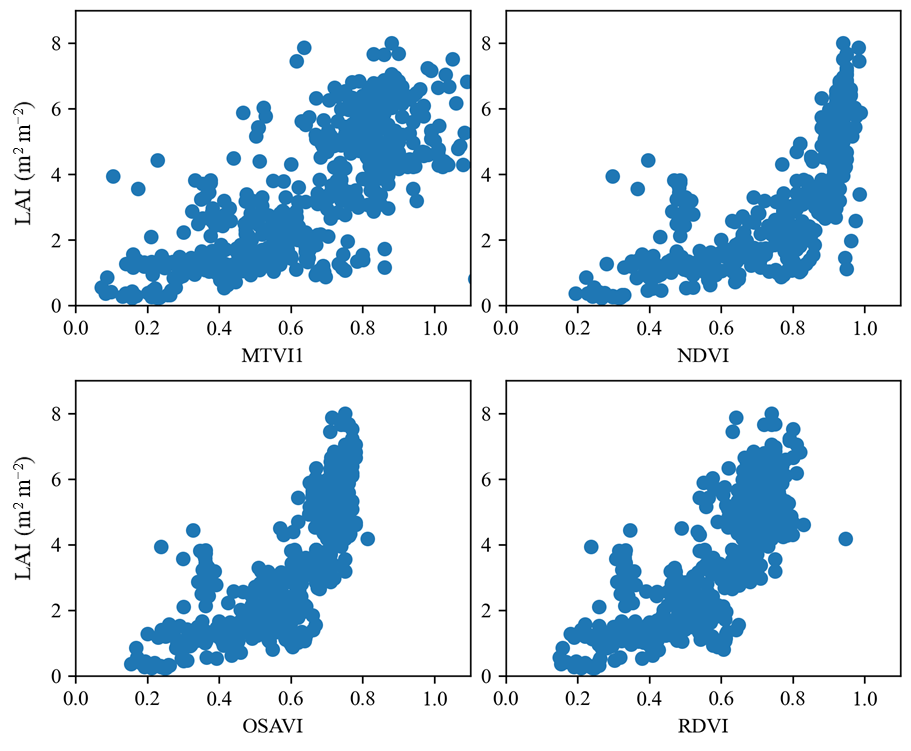


**Supplementary Figure 2.** Leaf area index (LAI) versus the vegetation indices (VIs) selected for soybean (n = 556) for the regression analyses in Table 1. The best regressor was Extra Trees, with a test score of 0.889. The VIs include the modified triangle vegetation index (MTVI), normalized vegetation index (NDVI), optimized soil adjusted vegetation index (OSAVI), and renormalized difference vegetation index (RDVI).


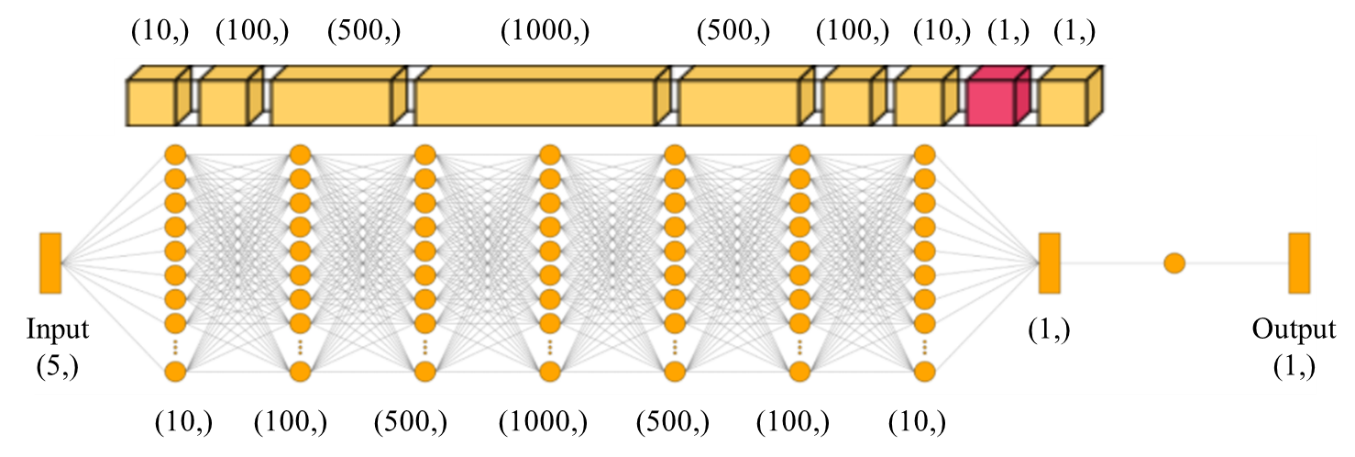


**Supplementary Figure 3.** Graphical representation of the deep neural network model structure applied in this study.


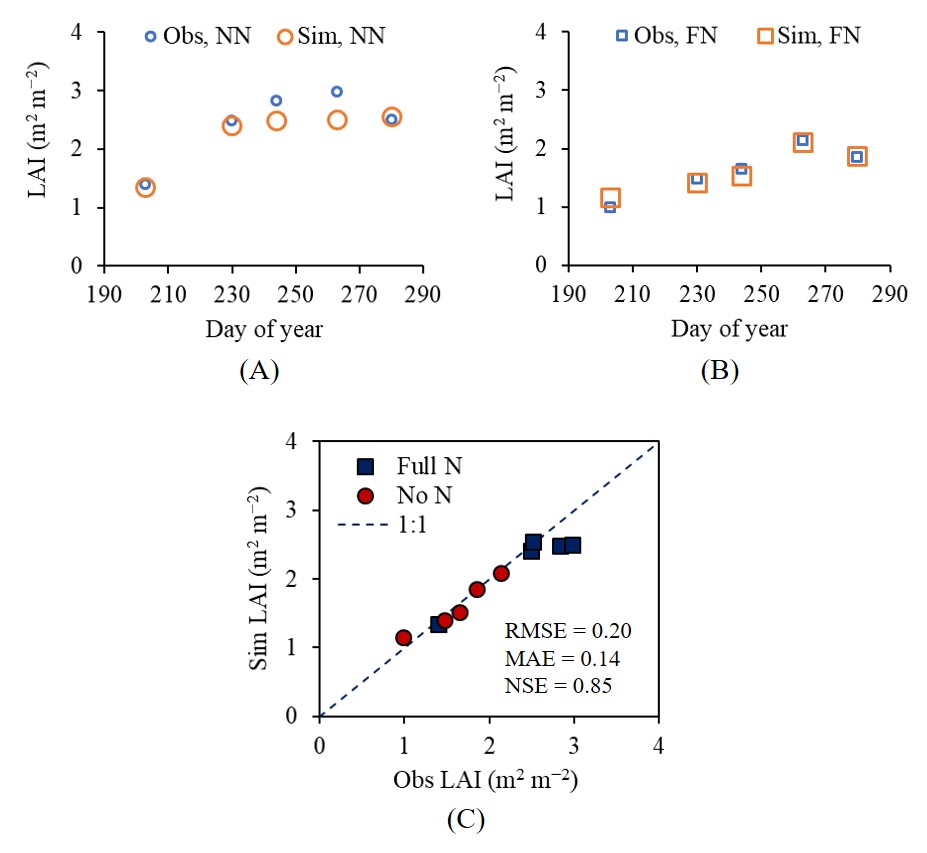


**Supplementary Figure 4.** Simulated (Sim) versus observed (Obs) leaf area index (LAI) values for rice grown with different nitrogen (N) treatments at the National Institute of Crop Science’s experimental field in 2022. Seasonal variations in the Sim and Obs LAI values with (A) no nitrogen (NN) and (B) full nitrogen (FN) treatments are shown along with (C) a comparison between the Sim and Obs LAI values including both N treatments. The diagonal dashed reference line in (D) represents the 1:1 relationship, and the root mean square error (RMSE), mean absolute error (MAE), and Nash–Sutcliffe efficiency (NSE) values for the predictions are displayed.


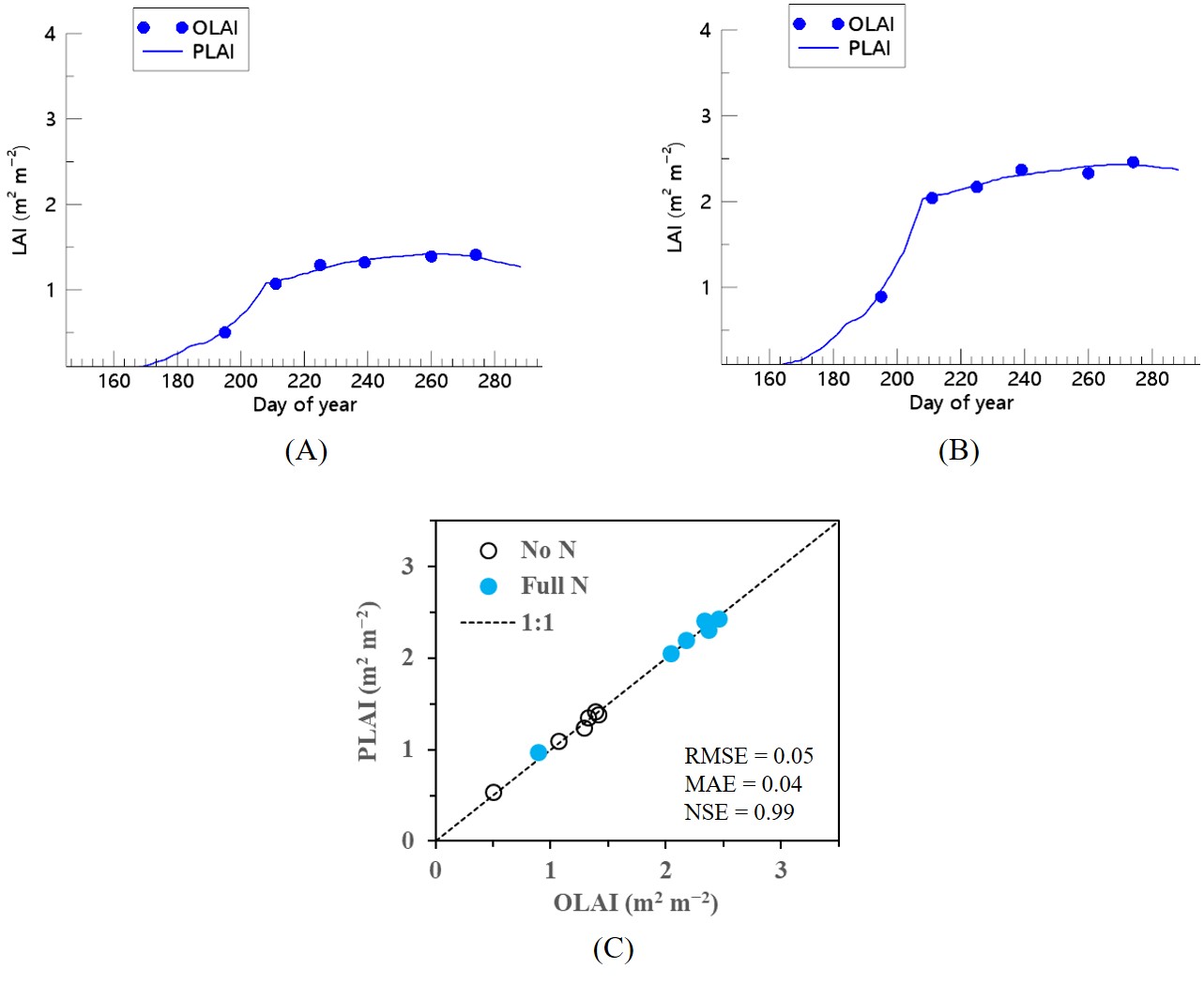


**Supplementary Figure 5.** Predicted (PLAI) versus observed (OLAI) leaf area index (LAI) values of rice grown with different nitrogen (N) treatments at the National Institute of Crop Science’s experimental field in 2021. Seasonal variations in LAI values with (A) no N and (B) full N treatments are shown along with (C) a comparison between PLAI and OLAI including both N treatments. The diagonal dashed reference line in (D) represents the 1:1 relationship, and the root mean square error (RMSE), mean absolute error (MAE), and Nash–Sutcliffe efficiency (NSE) values for the predictions are displayed.


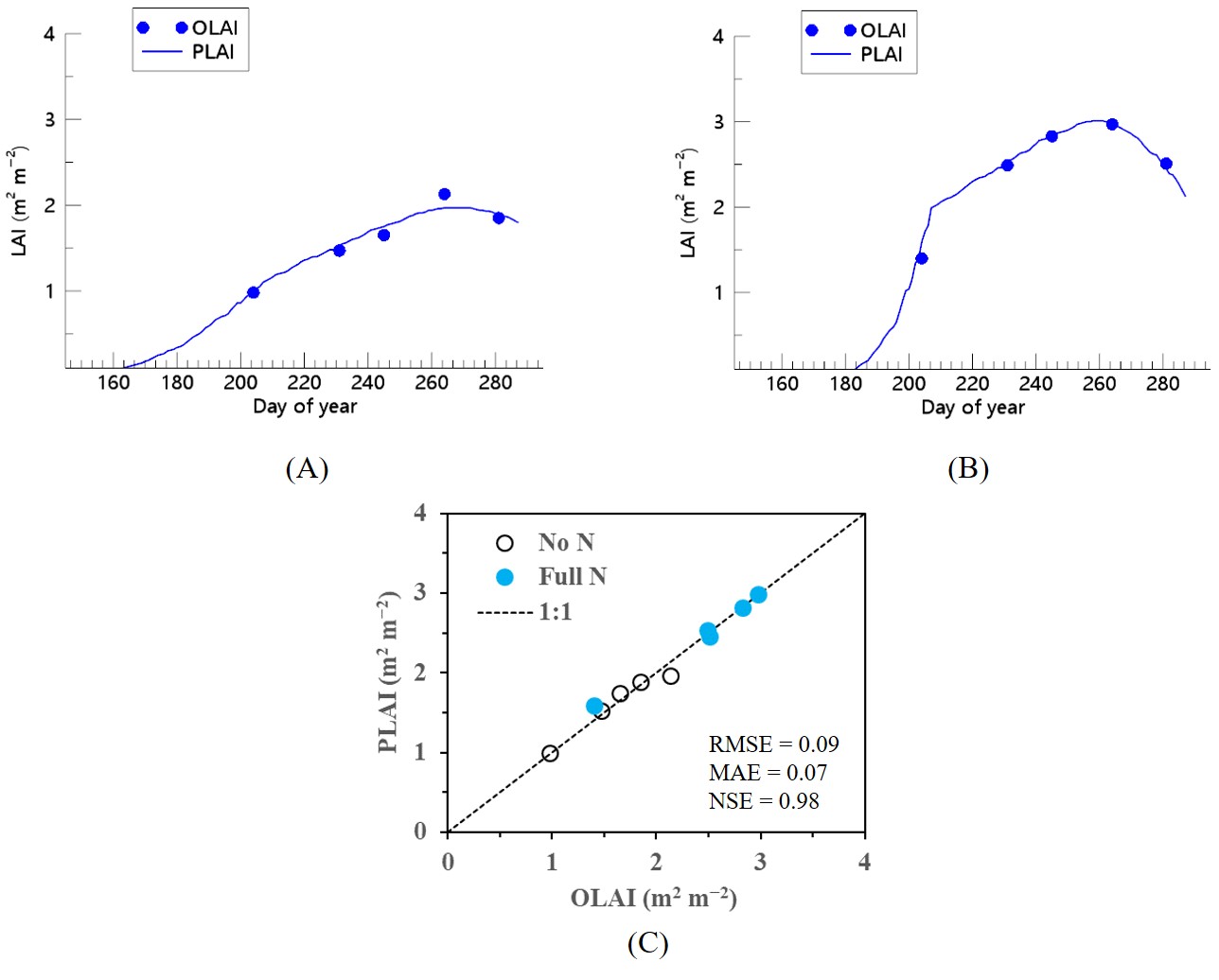


**Supplementary Figure 6.** Predicted (PLAI) versus observed (OLAI) leaf area index (LAI) values of rice grown with different nitrogen (N) treatments at the National Institute of Crop Science’s experimental field in 2022. Seasonal variations in LAI values with (A) no N and (B) full N treatments are shown along with (C) a comparison between PLAI and OLAI including both N treatments. The diagonal dashed reference line in (D) represents the 1:1 relationship, and the root mean square error (RMSE), mean absolute error (MAE), and Nash–Sutcliffe efficiency (NSE) values for the predictions are displayed.

# **Supplementary Tables**

**Supplementary Table 1.** Exponential regression coefficients determined for the relationships between the VI and LAI values of rice and soybean using the data in Supplementary Figures 1 and 2.

| Crop | VI^†^ | LAI = a*exp(VI*b) | |
| --- | --- | --- | --- |
|  |  | a | b |
| Rice | MTVI1 | 0.34 | 3.74 |
|  | NDVI | 0.19 | 3.56 |
|  | OSAVI | 0.21 | 4.95 |
|  | RDVI | 0.25 | 4.85 |
| Soybean | MTVI1 | 0.62 | 2.49 |
|  | NDVI | 0.27 | 3.13 |
|  | OSAVI | 0.33 | 3.75 |
|  | RDVI | 0.41 | 3.55 |

^†^ MTVI1 = modified triangle vegetation index 1, NDVI = normalized vegetation index, OSAVI = optimized soil adjusted vegetation index, RDVI = renormalized difference vegetation index.

**Supplementary Table 2.** Converged parameters of initial leaf area index (*L_0_*) and leaf partitioning and senescence (*a, b,* and *c*) after the within-season calibration of the remote sensing-integrated crop model for the nitrogen (N) treatments of rice at Chonnam National University (CNU) and National Institute of Crop Science (NICS).

| Site | Year | Treatment | *L_0_* | *a* | *b* | *c* |
| --- | --- | --- | --- | --- | --- | --- |
| CNU | 2021 | No N | 0.0013 | 0.4687 | 0.0005 | 0.0033 |
|  |  | Basal N | 0.0088 | 0.5776 | 0.0004 | 0.0054 |
|  |  | Full N | 0.0054 | 0.5292 | 0.0004 | 0.0044 |
|  | 2022 | No N | <0.0001 | 0.0005 | 0.0095 | <0.0001 |
|  |  | Full N | <0.0001 | 0.0015 | 0.0078 | <0.0001 |
|  |  | Heavy N | <0.0001 | 0.0005 | 0.0080 | <0.0001 |
| NICS | 2021 | No N | 0.0140 | 0.6409 | 0.0003 | 0.0067 |
|  |  | Full N | 0.0131 | 0.5706 | 0.0004 | 0.0024 |
|  | 2022 | No N | 0.0024 | 0.6684 | 0.0002 | 0.0146 |
|  |  | Full N | <0.0001 | 0.1559 | 0.0012 | 0.0045 |

**Supplementary Table 3.** Converged parameters of initial leaf area index (*L_0_*) and leaf partitioning and senescence (*a, b,* and *c*) after the within-season calibration of the remote sensing-integrated crop model for the nitrogen (N) treatments of soybean at the National Institute of Crop Science (NICS).

| Year | Treatment^†^ | *L_0_* | *a* | *b* | *c* |
| --- | --- | --- | --- | --- | --- |
| 2021 | No N | 0.0733 | 0.3193 | 0.0008 | 0.0505 |
|  | N24 | 0.0307 | 0.1107 | 0.0015 | 0.0289 |
|  | N48 | 0.0851 | 0.3756 | 0.0007 | 0.0699 |
| 2022 | No N | 0.0423 | 0.0059 | 0.0035 | 0.0139 |
|  | N24 | 0.0567 | 0.0086 | 0.0033 | 0.0118 |
|  | N48 | 0.0417 | 0.0152 | 0.0030 | 0.0205 |

^†^ N24 = 24 kg N ha^−1^ and N48 = 48 kg N ha^−1^
